# Testicular impairment in Primary Adrenal Insufficiency caused by Nicotinamide Nucleotide Transhydrogenase (NNT) deficiency **- **a case report: implication of oxidative stress and importance of fertility preservation

**DOI:** 10.1186/s12610-022-00176-6

**Published:** 2023-03-14

**Authors:** Lucile Ferreux, Yasmine Boumerdassi, Emmanuel Dulioust, Xavier Bertagna, Florence Roucher-Boulez, Mathilde Bourdon, Nicolas Thiounn, Catherine Patrat

**Affiliations:** 1 Service de Biologie de La Reproduction-CECOS, CHU Cochin – Bâtiment Port Royal, Hôpitaux de Paris (AP- HP), APHP. Centre – Université de Paris CitéHôpital Cochin, 123 Boulevard de Port-Royal, 75679 Paris 14, France; 2 Université de Paris Cité Institut Cochin, U1016, INSERM, CNRS, F-75014 Paris, France; 3 Hôpitaux de Paris (AP- HP), APHP. Centre – Université de Paris Cité, Hôpital Cochin, Service d’endocrinologie Et Maladies Métaboliques, Paris, France; 4 Hospices Civils de Lyon, HCL. Centre – Laboratoire de Biochimie Et Biologie Moléculaire, UM Endocrinologie- Centre de Référence du Développement Génital- Univ Lyon, Université Claude Bernard, Lyon 1, Lyon, France; 5 Hôpitaux de Paris (AP–HP), AP-HP. Centre – Université de Paris Cité, Hôpital Cochin, Service de Gynécologie-Obstétrique II Et de Médecine de La Reproduction, Paris, France; 6 Hôpitaux de Paris (AP- HP), APHP. Centre – Université de Paris Cité, Hôpital Européen Georges-Pompidou (HEGP), Service d’Urologie, Paris, France

**Keywords:** NNT deficiency, Primary adrenal insufficiency, Oxidative stress, Obesity, Testicular adrenal rest tumors, Déficit NNT, Insuffisance surrénalienne primaire, Stress oxydatif, Obésité

## Abstract

**Introduction:**

*Nicotinamide nucleotide transhydrogenase* (*NNT*) gene deficiency has recently been shown to be involved in Primary Adrenal Insufficiency (PAI). *NNT* encodes an inner mitochondrial membrane protein that produces large amounts of NADPH. NADPH is used in several biosynthesis pathways and the oxidoreduction of free radicals by the glutathione and thioredoxin systems in mitochondria. Patients with PAI due to NNT deficiency may also exhibit extra-adrenal manifestations, usually including gonadal impairment.

**Case report:**

We present the case of a 35-year-old patient referred to our center for primary infertility with non-obstructive azoospermia, in a context of PAI and obesity. PAI genetic exploration carried out at the age of thirty revealed NNT deficiency due to the presence of two deleterious mutations (one on each allele) in the *NNT* gene. Scrotal ultrasound revealed a right Testicular Adrenal Rest Tumor (TART). Intensification of glucocorticoid therapy over the course of 8 months failed to reduce the TART volume or improve sperm production and endocrine function. No spermatozoa were found after surgical exploration of both testes, and subsequent histopathological analysis revealed bilateral Sertoli cell-only syndrome. A retrospective review of the hypothalamic-pituitary-gonadic axis hormonal assessment over 20 years showed progressive impairment of testicular function, accelerated during adulthood, leading to hypergonadotropic hypogonadism and non-obstructive azoospermia when the patient reached his thirties, while the PAI remained controlled over the same period.

**Conclusion:**

This case report provides, for the first time, direct evidence of complete germ line loss in an azoospermic man with NNT deficiency. Additional data further support the hypothesis of a determinant role of oxidative cellular damage due to reactive oxygen species (ROS) imbalance in the severe gonadal impairment observed in this NNT-deficient patient. Early and regular evaluation of gonadal function should be performed in patients with PAI, especially with NNT deficiency, as soon as the patients reach puberty. Fertility preservation options should then be provided in early adulthood for these patients.

## Résumé

**Introduction:**

Le gène *Nicotinamide Nucleotide Transhydrogenase* (*NNT*) a été récemment impliqué dans l’Insuffisance Surrénalienne Primaire (ISP). Il code pour une protéine de la membrane mitochondriale interne qui produit de fortes quantités de NADPH. Le NADPH est utilisé par plusieurs voies de biosynthèse et dans l’oxydo-réduction de radicaux libres par les voies de signalisation impliquant le glutathion et la thioredoxine dans la mitochondrie. Les patients avec une ISP, en lien avec un déficit du gène NNT, peuvent également présenter des manifestations extra-surrénaliennes, dont une altération gonadique.

**Cas clinique:**

Nous présentons le cas clinique d’un homme de 35 ans adressé à notre centre d’Assistance Médicale à la Procréation pour infertilité primaire avec azoospermie non obstructive, dans un contexte d’ISP et d’obésité. L’exploration génétique effectuée à l’âge de 30 ans a identifié un déficit complet de la protéine NNT dû à la présence de deux mutations hétérozygotes (une sur chaque allèle), délétères. L’échographie scrotale a montré une tumeur testiculaire d’origine surrénalienne à droite. L’intensification du traitement par glucocorticoides pendant 8 mois n’a pas réduit le volume de la tumeur ni amélioré la production spermatique ou la fonction testiculaire endocrine. Aucun spermatozoïde n’a été retrouvé après exploration chirurgicale testiculaire bilatérale, en lien avec un syndrome de Cellules de Sertoli Seules. L’étude rétrospective de l’axe hypothalamo-hypophysaire-gonadique monte une altération progressive de la fonction testiculaire, accélérée à l’âge adulte, aboutissant à un hypogonadisme hypergonadotrope et une azoospermie non-obstructive à 30 ans, alors que l’ISP était contrôlée pendant cette période.

**Conclusion:**

Ce cas clinique met en évidence pour la première fois une disparition complète de la lignée germinale chez un patient avec un déficit en NNT. Il avance des arguments en faveur de l’hypothèse d’un rôle déterminant des dommages cellulaires, dus à un excès de radicaux oxygénés dans cette atteinte régulière de la fonction gonadique. Cette dernière devrait être suivie à partir de la puberté chez les patients ISP et plus particulièrement ceux ayant un déficit en NNT. Une préservation de la fertilité pourrait leur être proposée lorsqu’ils deviennent adultes.

## Introduction

Primary adrenal insufficiency (PAI) comprises a rare but potentially life-threatening heterogeneous group of endocrine disorders [1]. It is characterized by impaired steroid hormone(s) secretion (glucocorticoid and/or mineralocorticoid) by the adrenal cortex. In children, PAI is mainly due to genetic defects, the most frequent being Congenital Adrenal Hyperplasia (CAH) caused by 21-hydroxylase deficiency (between 55 and 75%) [2]. The other cases are of autoimmune origin (~ 20%, including Addison’s disease), or are caused by other genetic defects (~ 20%) [3]. PAI usually manifests within the first days to weeks of life as dehydration or salt-wasting syndrome. Several clinical manifestations are related to cortisol deficiency, such as tiredness, feeding difficulties, diarrhea, vomiting, weight loss, and anorexia. Hypoglycemia may also be part of the clinical features and can lead to seizures. Alteration of renal function is also present, including hyponatremia, hyperkalemia, acidosis, and elevated plasma creatinine. A specific sign of PAI is hyperpigmentation of the skin and mucosal membranes, resulting from high proopiomelanocortin and corticotropin secretion in response to decreased cortisol levels [4].

In the past decade, the development of high throughput sequencing has accelerated the identification of new genetic causes of PAI [5, 6]. Some of these recently identified PAI genes are involved in various cellular processes, including DNA replication (*MCM4*), regulation of the cell cycle, nuclear protein import (*AAAS*), metabolism growth (*SAMD9*, *CDKN1C*, etc.), as well as defense mechanisms against oxidative cellular damage (*NNT*, *TXNRD2*, etc.). These latter findings suggest that the adrenal cortex may be hypersensitive to oxidative stress.

Mutations in the *nicotinamide nucleotide transhydrogenase* (*NNT*) gene were first described in FGD in 2012 [7]. The *NNT* gene is a highly conserved gene that is ubiquitously expressed in human tissues and includes 22 exons and encodes a protein of 1086 amino acids. This protein is located in the inner mitochondrial membrane, where it acts as a proton pump transhydrogenase that generates nicotinamide adenine dinucleotide phosphate (NADPH) [8]. The NNT protein comprises three domains. Domains I and III contain the hydrophilic NAD(H) binding site and the NADP(H) binding sites, respectively. Domain II is the transmembrane part of the enzyme that connects domains I and III and forms the proton channel [9]. The active form of the enzyme is a homodimer of ~ 220 kDa. Mitochondria are essential for steroid hormone synthesis but are also a major source of radical oxygen species (ROS) [10]. NNT produces high concentrations of NADPH for the detoxification of ROS by glutathione and thioredoxin systems in mitochondria, and NNT deficiency results in decreased NADPH production, which leads to widespread failure of the ROS detoxification systems throughout the organism. Forty homozygous or compound heterozygous mutations have been identified in *NNT* gene to date.

Aside from complete adrenal insufficiency, observed in approximately 30% of patients [8], the NNT mutations identified also induce extra-adrenal manifestations, such a cardiac dysfunction [11] and hypothyroidism [8]. Testicular dysfunction in a patient with an NNT mutation was first described by Hershkovitz et al. in 2015 [12]. In the cohort of 18 patients carrying NNT mutations described by Roucher-Boulez et al*.* in 2016, the testis was the most frequently affected organ (approximately 30%) after the adrenal cortex. Moreover, cryptorchidism, Testicular Adrenal Rest Tumors (TART), and precocious puberty were also reported [8].

In this report, we describe the progressive deterioration of testicular function over nearly twenty years, leading to definitive non-obstructive azoospermia, in a patient carrying two different NNT mutations (one on each allele). This patient was included in the cohort studied by Roucher-Boulez et al*.* [8]. We provide numerous additional data relevant to gonadal function and discuss the plausible involvement of oxidative damage linked to NNT deficiency in this progressive deterioration of spermatogenesis.

## Case presentation

A 35-year-old man was referred to our Assisted Reproductive Technology center (Fertilité Paris Centre, Cochin, APHP) for primary infertility for two years, in a context of azoospermia. The patient ‘s wife was 27 years old and her fertility assessment was normal. The patient’s prior medical history revealed PAI with combined mineralocorticoid and glucocorticoid deficiency discovered after an episode of acute dehydration when he was 9 months old. He is Caucasian, his parents are not consanguineous, and he has a healthy younger brother and sister and has no family history of infertility. The patient had no history of testicular trauma, cryptorchidism, or inguinal or scrotal surgery. He did not report professional exposure to heat or endocrine disrupting chemicals, nor tobacco, alcohol, or drug use. Physical examination revealed that the patient exhibited abdominal stretches and moderate skin hyperpigmentation. His height was 180 cm and his weight was 130 kg, with a Body Mass Index (BMI) of 40.1 kg/m^2^, indicating morbid obesity. Obesity has been reported since he was 4 years old and became permanent despite dietary follow-up. Puberty spontaneously occurred at the age of 13. The secondary sex characteristics were present, but the testes were hypotrophic. The vas deferens and epididymis were palpable on both sides and were normal.

During childhood and adolescence, the patient had received regular follow-up by pediatric endocrinologists and underwent steroid (glucocorticoid and mineralocorticoid) replacement therapy in addition to receiving adequate patient education on managing the illness and medication. The very long-chain fatty acid assessment was normal. The patient was initially screened for *DAX-1* and *SF-1* genes during their childhood, but no mutations were found. He was screened again in 2013, after new gene mutations had been identified in FGD (*MC2R*, *MRAP*, *StAR*, *AAAS*, *GPX1,* and *NNT*). The results revealed a mutation in exon 4 of the NNT gene, inherited from his father and responsible for a premature STOP codon p.Arg71* (NM_012343.3:c.211 C > T). A partial NNT deletion of exons 2 and 3 (c.(-51 + 1_-53–1)_ (381 + 1_382-1)del) was found on the maternal allele. To confirm the deletions, array comparative genomic hybridization (aCGH) was performed using an Agilent SurePrint G3 Human CGH Microarray 4 × 180 K AMADID 022,060 (Agilent Technologies) according to the manufacturer’s instructions. This procedure was followed by long-range PCR using a Qiagen LongRange PCR Kit (Qiagen) according to the supplier’s recommendations. Conventional dideoxy sequencing of the PCR product was undertaken (primers available on request).

A first semen analysis performed when the patient was 35 years old revealed azoospermia, which was confirmed by a second semen analysis performed in our center, with normal volume (3.6 mL) and pH (7.7). His seminal biochemical markers were normal. A scrotal ultrasonography confirmed testicular hypotrophy (left: 8 mL, right: 12 mL), with bilobular calcifications on both sides (left: 13 mm; right: 7 mm). No vascular anomalies were observed on Doppler analysis. A hypoechogenic lesion of 8 × 5 mm was observed on the anterior region of the right testis, suggesting, at first sight, a Testicular Adrenal Rest Tumor (TART). In July 2013, the hypothalamic-pituitary–gonadal axis was explored and revealed elevated gonadotrophin levels (FSH: 19 IU/L, NR: 1–6, LH: 12 IU/L, NR: 0.8–6), with a low serum total testosterone level (5.82 nmol/L, NR: 11.8–34.5) and a normal SHBG level (11.9 nmol/L, NR: 17.3–65.8), consistent with hypergonadotropic hypogonadism. The inhibin B level was also low (14 pg/mL, NR: 28–267), revealing altered Sertoli cell function. The patient’s karyotype was normal (46, XY), and no partial deletion was found in the AZF locus of the Y-chromosome. Regarding the corticotropic axis hormonal assessment, the ACTH level was high (198.9 pmol/L, NR: < 13.3), with an undetectable morning serum cortisol level. Adrenal androgen levels were low (∆4-androstenedione: 0.72 nmol/L, NR: 2.8–7.4; DHEA-sulfate: 0.2 µmol/L, NR: 2.4–13.4), 17-OH-progesterone was 1.48 nmol/L (NR: 4.5–8.5), and the thyroid function was normal (TSH: 3.0 IU/L, NR: 0.4–4.5) (Table 1). Aside from elevated levels of ACTH, the PAI appeared stable, with no acute crisis reported during adulthood, as well as normal renal function under appropriate treatment and with good therapeutic compliance. The patient was advised to lose weight and was referred to a dietician team.

**Table 1:** Selected hormonal values of the patient between 1997 and 2019

	**FSH (IU/L)**	**LH (IU/L)**	**Serum total Testosterone (nmol/L)**	**Inhibin B (pg/mL)**	**ACTH (pmol/L)**	**Cortisol (nmol/L)**
**Normal range**	1–6	0.8–6	11.8–34.5	28–267	< 13.3	166–507
**DATE**						
**September-1997**	4	5	13,18		108,3	< 28
**October-1999**	5,2	7	11,09		166,3	< 28
**September-2001**	4,8	7,8	4,51		916,1	< 28
**November-2002**	5,5	11,6	9,36		867,7	28
**May-2004**	4,9	9,7	7,28		231,4	< 28
**April-2008**	5,6	7	12,14		118,3	
**May-2010**	5,5	8,6	11,09		1000	28
**July-2013**	19	12	5,82	14	198,9	< 28
**March-2014**	28	13	6,24		2,2	
**March-2015**	30,8	10,2	4,89		2	
**September-2017**	2,4	0,66	5,91		566	< 4
**October-2019**	0,5	0,5	7,9		155	< 4

Serum total testosterone was stable until 2010 (31 year-old), before decreasing, with higher gonadotrophin levels. ACTH levels were high except during the intensified glucocorticoid therapy (between August 2013 and March 2015)

*ACTH* Adrenocorticotropic hormone, *FSH* Follicle stimulating hormone, *LH* Luteinizing hormone, *IU* international unit, *nmol/L* nanomoles per liter, *pg/mL* picograms per milliter, *pmol/L* picomoles per liter

**Fig. 1:**
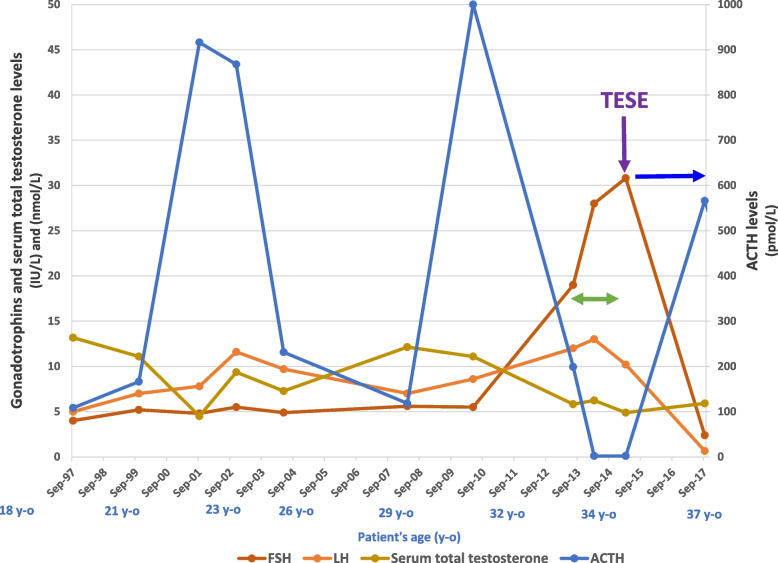
Changes in the hypothalamic-pituitary-axis and glucocorticoid axis between 1997 and 2017. The glucocorticoid axis is represented by ACTH levels. Higher ACTH levels are observed in the patient’s early twenties and thirties, which can be explained by the patient’s lack of compliance with medication and dietary measures during this period. The ACTH levels were undetectable during intensified glucocorticoid therapy (green arrows). The serum total testosterone levels started to decrease in the patient’s thirties, associated with higher gonadotrophin levels. The intensified glucocorticoid therapy did not contribute to an increase in the serum total testosterone levels. After TESE (purple arrow), an androgen substitution therapy was introduced (blue arrow), which led to a negative feedback on the pituitary–gonadal axis, with low gonadotrophin and serum total testosterone levels. ACTH: Adrenocorticotropic hormone, FSH: Follicle-stimulating hormone IU/L: international units per liter, LH: Luteinizing hormone, nmol/L: nanomole per liter, pmol/L: picomoles per liter, TESE: Testicular sperm extraction, y–o: year-old

After consultation with the endocrinology and urology teams, the patient was offered intensified glucocorticoid treatment, as a number of studies have reported improvement of semen parameters in patients with TART [13, 14]. Dexamethasone (0.5 mg per day) was added to the steroid replacement therapy (hydrocortisone: 20 mg per day; fludrocortisone: 150 µg per day). After 18 months of follow-up, the patient’s BMI was stable (40.1 kg/m^2^). The hormonal assessment showed lower ACTH levels (2.2 pmol/L, NR: < 13.3) after intensified glucocorticoid therapy, but persistently elevated levels of gonadotrophins (FSH: 30.8 IU/L, NR: 1–6; LH: 10.2 IU/L, NR: 0.8–6) and low total serum testosterone levels (4.89 nmol/L, NR: 11.8–34.5) Fig. 1, Table 1. The dexamethasone had no impact on the TART, with a stable volume of the right testicle lesion (7 × 6 mm) Table 2. A semen analysis was also performed and confirmed the persistent azoospermia.

**Table 2:** Scrotal ultrasonography results

**Date**	**Right Testicle (mL)**	**Left Testicle (mL)**	**Right TART (mm)**
**April 2013**	12	8	8*6
**September 2013**	10	8	8*5
**March 2014**	10	8	9*5
**March 2015**	10	7	7*6

Scrotal ultrasonography revealed a bilateral testicular hypotrophy, and a TART localized in the right testicle. Despite the intensified glucocorticoid therapy (between August 2013 and March 2015), the TART size remained stable

*mL* milliliter, *mm* millimeter, *TART* Testicular Adrenal Rest Tumor

After multidisciplinary consultation, the medical team recommended performing surgical testicular sperm extraction (TESE), while informing the patient of the low probability of finding spermatozoa and the requirement for testosterone substitution after surgery. The patient provided his informed consent, and bilateral testicular pulp extraction was performed on February 2015, but no spermatozoa were found. Histopathological analysis revealed a complete loss of germ line cells, with a Sertoli cell-only pattern. The interstitium of the right fragment contained islets of hyperplasic Leydig cells, with no neoplastic signs. The patient started testosterone replacement therapy after surgery (testosterone enanthate 250 mg IM, every 3 weeks). The couple accepted sperm donation, and they became parents of a healthy boy in 2017, after three cycles of intra-uterine insemination with donor sperm.

## Discussion

This report describes the case of a man monitored since early infancy for Primary Adrenal Insufficiency (PAI) caused by NNT deficiency diagnosed at the age of thirty, after the discovery of non-obstructive azoospermia.

Based on the available data from the patient’s follow-up between 18 and 37 years of age, the diagnostic explorations of his infertility, and the mutations identified in the *NNT* gene,three non-exclusive hypotheses were considered to explain the azoospermia.

The first hypothesis was the presence of a TART in the right testis. TARTs are typically located in the rete testis and, by compressing the tubules of the rete testis, can inhibit transit of spermatozoa towards the epididymis. TARTs were initially described in patients with CAH and are observed in patients with PAI [15]. TART usually regress within the first year of life, except if its growth is stimulated by high serum ACTH levels because adrenal rest cells retain ACTH receptors [16]. Several reports have described a decrease in tumor size after intensified glucocorticoid therapy, which led to lower ACTH levels [14, 17–19]. However, TARTs are also found in well-controlled PAI patients, which suggests that other factors may also be involved [20]. TARTs may also lead to the destruction of normal tissue, as some patients with 21-hydroxylase deficiency and TARTs have significantly higher mean FSH levels, lower mean inhibin B levels, and lower sperm counts than patients without TART [14, 17–19]. However, for our patient, implication of the TART in the azoospermia appeared unlikely: as a unilateral TART could not itself account for the azoospermia nor for the similar histological pattern of atrophic seminiferous tubules with the complete loss of germ line cells observed in both testicles. These discrepancies suggested another cause for the testicular damage in this patient.

Another factor that could explain the patient’s infertility was the persistent obesity that the patient had exhibited since childhood. Obesity is currently recognized as one of the main factors of male infertility, inducing quantitative and qualitative alterations of sperm production [21–26]. Sperm quality alteration caused by obesity can be explained by several frequently associated mechanisms, such as hormonal imbalance, chronic inflammatory condition, scrotal hyperthermia, accumulation of toxic substances in adipocytes, and oxidative stress. Thus, obesity may have played an aggravating role in the deterioration of testicular function in our patient.

A third hypothesis to explain the azoospermia due to a complete loss of germline cells in our patient was the accumulation over the years of oxidative damage in the testicular tissue. This hypothesis is supported by several arguments.

NNT catalyzes a complex reaction that leads to the production of NADPH. It provides approximately 50% of the amount of NADPH necessary for the detoxification, by the glutathione and thioredoxin systems, of the ROS generated by mitochondrial respiration [27, 28]**.** NNT-mutated C57BL/6 J mice, in which a deletion in the NNT gene results in nearly no NNT activity, exhibit major alterations in the mitochondrial redox balance, such as increased release of H_2_O_2_, spontaneous oxidation of NADPH, and an increased oxidized/reduced glutathione ratio. These observations confirm that the absence of functional NNT results in severe deterioration of redox homeostasis that is not offset by the other sources of NADPH [29]. Another interesting study investigated the consequences of inactivation of the *PRX4* (Peroxiredoxin 4) gene in C57BL/6 J mice [30]. *PRX4* belongs to a family of enzymatic proteins involved in antioxidant defense and redox signaling and is highly expressed in testis [27]. A gene array analysis performed on rat gonocytes and spermatogonia confirmed that the Peroxiredoxins system plays a major role in the antioxidant defense system in these cells [31]. Interestingly, *PRX4* reduces peroxides and, in turn, its oxidized form is reduced and reactivated by a thiol-dependent process driven by thioredoxins and glutathione systems whose activity requires NADPH, known to be deficient in case of NNT mutation [32]**.** A testicular atrophy was observed in maturing and PRX4 adult KO males compared to control mice, whereas their body weights were not different and no anomalies were seen in other organs. Analyses of this atrophy and its causes revealed several histological and cellular differences with the control males: reduced diameter of the seminiferous tubules; increased prevalence of degenerating germ cells and of TUNEL-positive cells, indicating increased levels of DNA fragmentation; and increased levels of oxidized stress biomarker 8 hydroxyguanosine (8-OHdG) in degenerating germ cells. Of note, these differences with the control males were amplified after in vivo exposure to a brief warming of their testes.

Juxtaposed with each other, the observations described above suggest a consistent succession of events leading from the absence of NNT function to degeneration of germ cells, eventually resulting in disappearance of the entire germ line: NNT deficiency causes reduced NADPH production, which induces dysfunction of critical redox activities; this dysfunction results in a chronic excess of ROS; oxidative damage accumulates over time in germ cells including spermatogonia, leading to their degeneration.

As a result of the regular hormonal assessment of the patient, we were able to record data on the hypothalamic-pituitary–gonadal axis until the patient was 31 years old (Fig. 1). During the study period, their body weight was stable and the PAI remained controlled, with undetectable levels of ACTH after intensified glucocorticoid therapy. The serum total testosterone was stable until the patient was 31 years old, and then rapidly decreased in three years, with higher gonadotrophin and lower total testosterone plasma levels. These variations can be considered to be a reflection of a progressive increase in testicular damage in a context of NNT deficiency, highlighting the importance of early and regular follow-up of gonadic function in PAI. Moreover, it seems like the low testosterone levels observed during the last five years, in contrasts with the increase in gonadotropin plasmatic level, reflects an impairment of Leydig cells function. It is interesting to note that contrary to germ cells, Leydig cells instead of degenerating and disappearing, showed a trend to hyperplasia. This important difference may be due to the fact that distinct impairment mechanisms predominate in each of these two cell types. To date, the pathophysiology of the abnormal gonadal phenotypes profile observed in patients with NNT loss of function mutations have yet to be elucidated [33]**.**

All the available biochemical, experimental, and clinical data leads us to believe that, in the present case, severe and chronic oxidative stress in the germ cells was the main factor responsible for the azoospermia [33]. We cannot, however, rule out an aggravating effect of obesity, probably by participating itself to oxidative imbalance. Either way, we suggest that, in this patient, the azoospermia may have been preceded by a period of a few years during which mature sperm production was present.

One limitation of this description is the lack of semen analysis data for this patient after the onset of puberty. However, it can be estimated that the 20-year follow-up of gonadotrophin levels available in this case provided a reliable indication of the degree of impairment of spermatogenesis and its acceleration in the later period. Another limitation of our study is the absence of data in humans similar to those obtained in mice, objectifying oxidative damage in tissue and testicular cells.

This case report emphasizes the importance of regular evaluation of gonadic function in all patients with PAI, especially for patients with NNT deficiency for whom gonadal dysfunction appears to be accelerated. For example, a semen analysis and scrotal ultrasonography should be performed in post-pubertal adolescents. Fertility preservation options, sperm freezing, or testicular sperm extraction, should be proposed in early adulthood as soon as semen parameters are altered. Furthermore, the impact on ovarian function in case of NNT mutation in women should be assessed, as NNT is also expressed in ovarian tissue.

## Conclusion

This case highlights that a specific genetic diagnosis of Primary Adrenal Insufficiency can induce fertility monitoring of patients, as *nicotinamide nucleotide transhydrogenase* mutation appears to have accelerated testicular damage. Several studies have shown the importance of the thioredoxin/peroxiredoxin system in the testis, and higher infertility rates are observed in patients exhibiting a redox imbalance. *Nicotinamide nucleotide transhydrogenase* deficiency could be a good model to explore the consequences of redox imbalance in testicular tissue. Finally, this case encourages taking into account a comprehensive view of the long-term effects of combined environmental and genetic factors and suggests that one model of azoospermia can obscure another.

## Data Availability

Data available on request.

## Abbreviations

aCGH: Array comparative genomic hybridization
ACTH: Adrenocorticotropic hormone
BMI: Body Mass Index
CAH: Congenital adrenal hyperplasia
CMA: Chromosomal microarray
FGD: Familial glucocorticoid deficiency
FSH: Follicle-stimulating hormone
GSH: Glutathione system
IU/L: International units per liter
LH: Luteinizing hormone
mL: Milliliter
mm: Millimeter
NAPDH: Nicotinamide adenine dinucleotide phosphate
NNT: Nicotinamide nucleotide transhydrogenase
nmol/L: Nanomoles per liter
NR: Normal range
PAI: Primary adrenal insufficiency
pg/mL: Picograms per milliliter
pmol/L: Picomoles per liter
PRDX: Peroxiredoxin
PUFA: Polyunsaturated fatty acids
ROS: Reactive oxygen species
TART: Testicular adrenal rest tumor
TESE: Testicular sperm extraction
SHBG: Sex hormone-binding globulin
8-OHdG: 8 Hydroxyguanosine
